# Rodent faunas from the Paleogene of south-east Serbia

**DOI:** 10.1007/s12549-017-0305-0

**Published:** 2017-11-10

**Authors:** Hans de Bruijn, Zoran Marković, Wilma Wessels, Miloš Milivojević, Andrew A. van de Weerd

**Affiliations:** 1 Department of Earth Sciences, Utrecht University, Heidelberglaan 2, 3584CS Utrecht, the Netherlands; 2 Natural History Museum in Belgrade, Njegoševa 51, Belgrade, 11000 Serbia

**Keywords:** Mammalia, Rodentia, Eocene, Oligocene, south-east Serbia

## Abstract

Seven new rodent faunas are described from the Pčinja and Babušnica-Koritnica basins of south-east Serbia. The geology of the Tertiary deposits in the Pčinja and Koritnica-Babušnica basins of south-east Serbia is briefly reviewed. The fossil content of the new vertebrate localities is listed, and an inventory of the rodent associations is presented. The rodent associations are late Eocene-early Oligocene in age, interpreted on biostratigraphical grounds. These are the first rodent faunas of that age from the Balkan area, an important palaeogeographic location between Europe and Asia. The Muridae, with the subfamilies Pseudocricetodontinae, Paracricetodontinae, Pappocricetodontinae, Melissiodontinae and ?Spalacinae, are dominant with eight genera, four of which are new. The diversity of the Melissiodontinae and Paracricetodontinae in the faunas suggests that these subfamilies originated in this region. The bi-lophodont cheek teeth occurring in the Oligocene assemblages are identified as the first record of the Diatomyidae outside of Asia. In light of the large amount of new data, the palaeogeographic setting and faunal turnover of the Eocene-Oligocene is discussed.

## Introduction

**Fig. 1:**
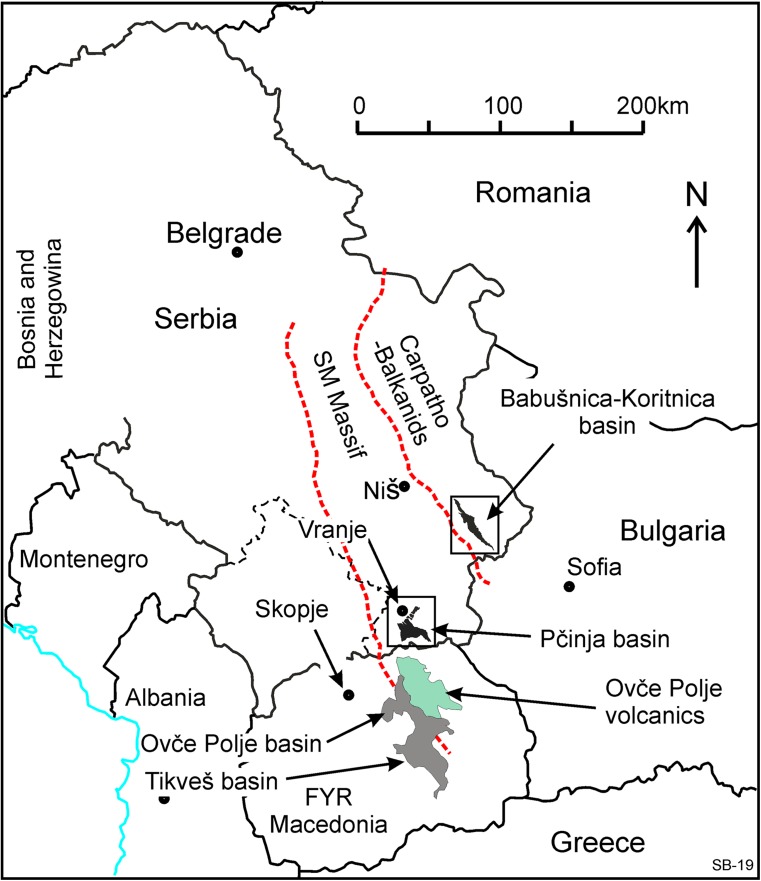
Location map of the Babušnica-Koritnica and the Pčinja Basins in Serbia. The broken red lines are the boundaries of the Serbian-Macedonian Massif

**Fig. 2:**
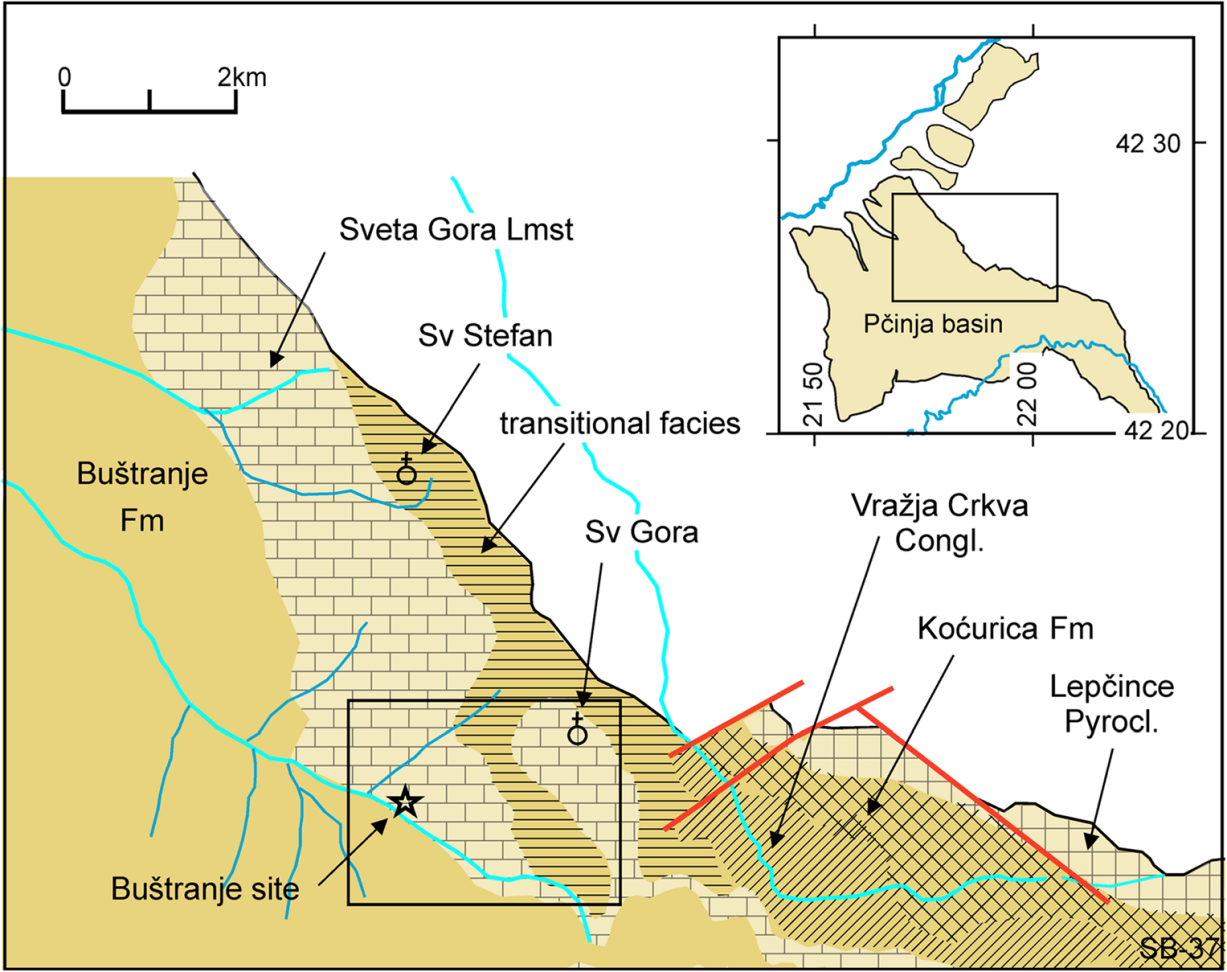
Map of the northern part of the Pčinja basin. Eocene deposits are in brown. Square refers to the satellite image of Fig. 3. Inset map shows the outline of the Pčinja basin (redrawn after Tercin et al. 1977; Dimitrijević and Dimitrijević 1987)

The Natural History Museum in Belgrade is carrying out a research program on Tertiary mammal faunas of the Balkan, and several new faunas have been found, described and published (e.g. the late Oligocene of the Banovići basin, de Bruijn et al. 2013, and the lower Miocene of the Levač basin, see Marković and Milivojević 2016). The Western and Central European Tertiary mammalian faunal history is relatively well known, and recently, many new data became available from Turkey and sites further afield in Asia. Information from the Balkan area is crucial because of its location between Asia and Central Europe. In their search for new data, Zoran Marković and Miloš Milivojević (Natural History Museum in Belgrade) discovered the first remains of Paleogene mammals during a reconnaissance trip in the spring of 2010 in Southern Serbia. These were found in a roadside exposure in the Babušnica-Koritnica basin (Fig. 1) near the village of Strelac. The first few rodent teeth from the fluvio-lacustrine sediments of this locality, now Strelac-1, shed light on a hitherto unknown assemblage of probably early Oligocene age. Several major reconnaissance and collecting campaigns were organised in continuing cooperation with Hans de Bruijn and Wilma Wessels (Utrecht University, the Netherlands). The Paleogene deposits of the Babušnica-Koritnica and Pčinja basins were sampled during the years 2010 to 2016. This joint action led to the discovery of more localities in the Babušnica-Koritnica basin yielding predominantly small mammal remains. Five of these are situated in the fluvio-lacustrine deposits of the Strelac area: Strelac-1, Strelac-2, Strelac-3, Valniš and Raljin and one in possibly turbidite deposits in the village of Zvonce. Later, Zoran Marković and Miloš Milivojević, prospecting the Pčinja basin near the town of Vranje (Figs. 1 and 2) for fossil mammals, found a locality of presumably (late?) Eocene age south-east of the village of Buštranje.

The aim of this paper is to make the general information about the location, content in terms of rodents and geological setting of the fossil sites in the Pčinja and Babušnica-Koritnica basins available. Since the rodent assemblages from the majority of the localities are quite diverse and contain many new species, the systematic palaeontology of the various (sub)families will be presented in separate future papers*.*

## Methods

The assemblages were collected by wet-screening fossiliferous matrix on a set of stable sieves (finest mesh used is 0.5 mm) in the field. The matrix from the localities Strelac, Zvonce and Buštranje had to be soaked in diesel fuel after drying before it would disintegrate in water. Except for the locality Strelac-1, the overburden covering the fossiliferous beds could be removed by a digging machine, which allowed taking large samples. The approximate weight of the samples processed from each locality is between 900 and 5000 kg per locality. The residues obtained from screen washing in the field have been rewashed on a set of vibrating sieves (Marković and Milivojević 2010). In order to reduce the lime-rich concentrates further, these were treated with diluted acetic acid in the laboratory. The gypsum crystals present in the residue from Raljin have been removed by heating the residue to 150° centigrade for several hours.

All specimens are shown in figures as left ones. If the original tooth is from the right side and figured as a left, its letter on the figures is underlined. Lowercase letters refer to the lower dentition, uppercase letters refer to the upper dentition.

The fossil assemblages from south-eastern Serbia are housed in the Natural History Museum in Belgrade (Serbia). A representative set of casts of rodents is kept in the collection of the Department of Earth Sciences of Utrecht University, the Netherlands. The codes and abbreviations used for the localities in the Natural History Museum Belgrade are as follows: 024 for Strelac-1 (STR1), 025 for Strelac-2 (STR2), 026 for Strelac-3 (STR3), 027 for Valniš (VA), 028 for Raljin-2 (RA2), 031 for Buštranje (BUS) and 036 for Zvonce (ZV). The figured specimens from Belgarite (BEL) are in the collection of Department of Earth Sciences, Utrecht University.

## Geological setting

### Pčinja basin

The basin is located close to the border with Macedonia within the Serbo-Macedonian Massif (Fig. 1; Dimitrijević 1997). The Serbo-Macedonian Massif is composed of metamorphic crystalline complexes of Proterozoic and Palaeozoic age; it is largely covered by Tertiary sediments. The Pčinja basin extends from the Vranje-Bujanovac trough in the NW into the Pčinja River valley in the SE over a distance of about 14 km (Fig. 2; Terzin et al. 1977). It is filled with poorly exposed Eocene and possibly Oligocene sediments.

In the Pčinja basin, these sediments overlie Proterozoic crystalline rocks, Proterozoic and Palaeozoic granites and Senonian rocks at places. The total basin fill may be up to 1500 m thick. Short descriptions of the lithological units of the Pčinja basin are in Dimitrijević and Dimitrijević (1981) and in Dimitrijević (1997); a detailed description is given in Dimitrijević and Dimitrijević (1987). A map of the northern part of the basin is shown in Fig. 2.

At the base of the basin fill is locally a very thick unit of subaerially deposited pyroclastics (Lepčince pyroclastic) followed by a succession of pyroclastics with intercalated conglomerates deposited in ephemeral and braided streams (Koćurice Fm). This grades upward into non-marine clastics (conglomerates, sandstones, siltstones, clays) of the Vražja Crkva Fm. A deepening of the depositional environment resulted in the deposition of a turbiditic succession of up to 120 m thickness (Buštranje Fm), at many places slumped and followed by 120 m of the Kukavica turbidites. Marine fossils have not been described from this succession, but at places, plant fossils have been found. A unit of freshwater limestone interbedded with marly argillaceous beds (Sveta Gora Fm) up to 100 m thick is developed near the northern margin of the basin. This unit is probably lateral of the Buštranje Formation.

Dimitrijević and Dimitrijević (1981, 1987) suggested that the Pčinja area was part of a basin covering a large part of Macedonia and included the Ovče Pole and Tikveš basins during the Eocene-Oligocene (Fig. 1). Brief descriptions of the Eocene-Oligocene basin fill of Ovče Pole (=Ovchepole) and Tikveš are in Dumurdzanov et al. (2004) and Stojanova and Petrov (2012). The total thickness of the basin fill is 3500–4000 m; the basal coarse clastics 600–1000 m are undated, most of the basin fill is of Priabonian age; and the upper 300–400 m of the section is Oligocene. Two thick turbiditic intervals (each 1200–1300 m thick) are present, both Priabonian in age, and these have been linked to the turbiditic units of the Pčinja basin by Dimitrijević and Dimitrijević (1981, 1987).

Fossil plants described by Mihajlović (1985) from the Buštranje Formation suggest an Eocene-Oligocene age; Dimitrijević and Dimitrijević (1981, 1987) assume a Priabonian to early Oligocene age. The plant localities 1–6 of Mihajlović are in the Pčinja River valley and given a late Eocene Age. His sites 7–8 are in or near the Žbevac Sandstone Formation of the Pčinja basin (not discussed in this paper) and considered Oligocene in age. Marine micro-fossils (dinoflagellates) from an unspecified location or stratigraphic interval have been mentioned by Mihajlović (1985, p. 422).

#### The Buštranje fossil mammal locality in the Pčinja basin

**Fig. 3:**
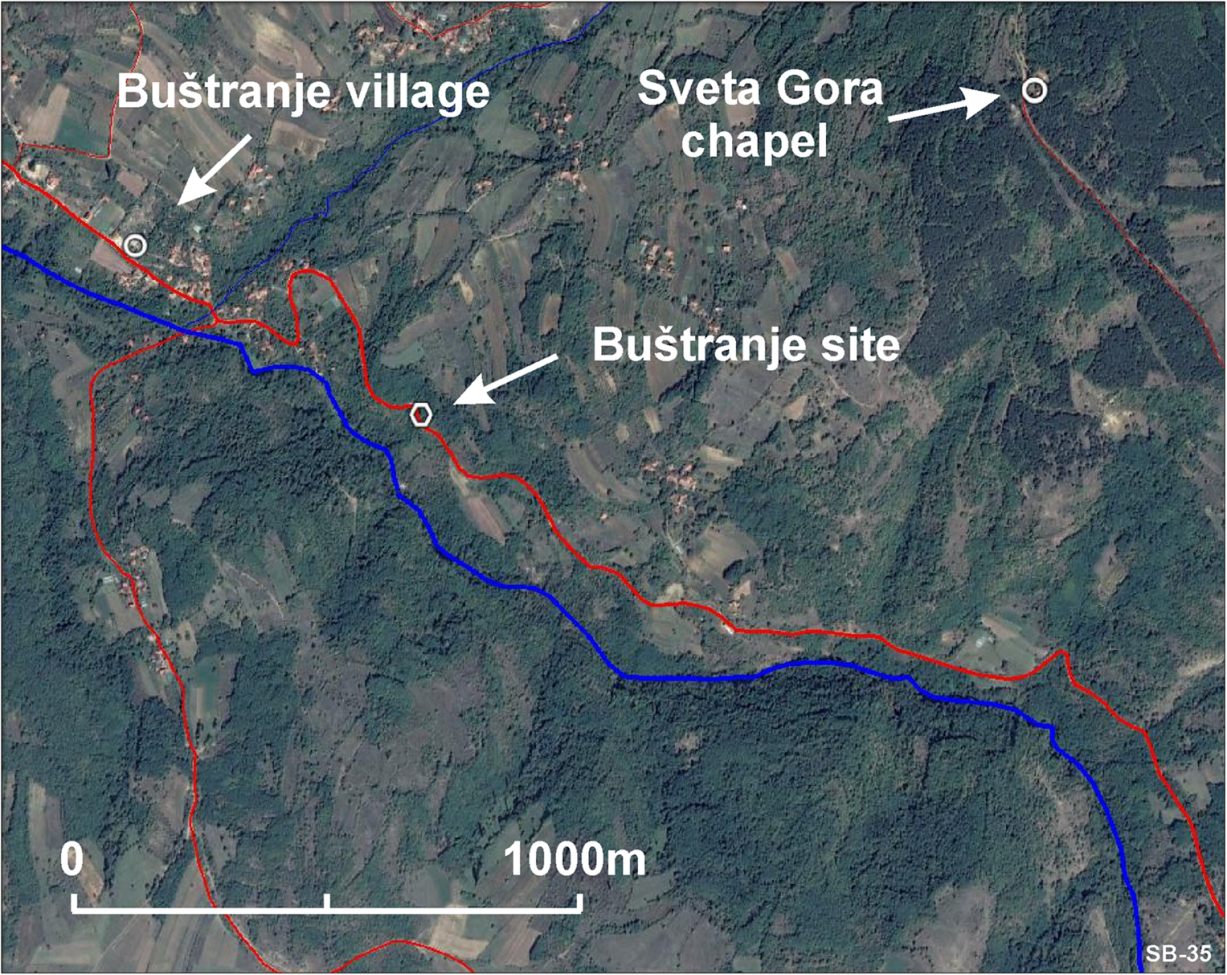
Satellite image showing the location of the Buštranje locality. The road is in red (satellite image copyrights Google 2017; image CNES/Airbus)

**Fig. 4:**
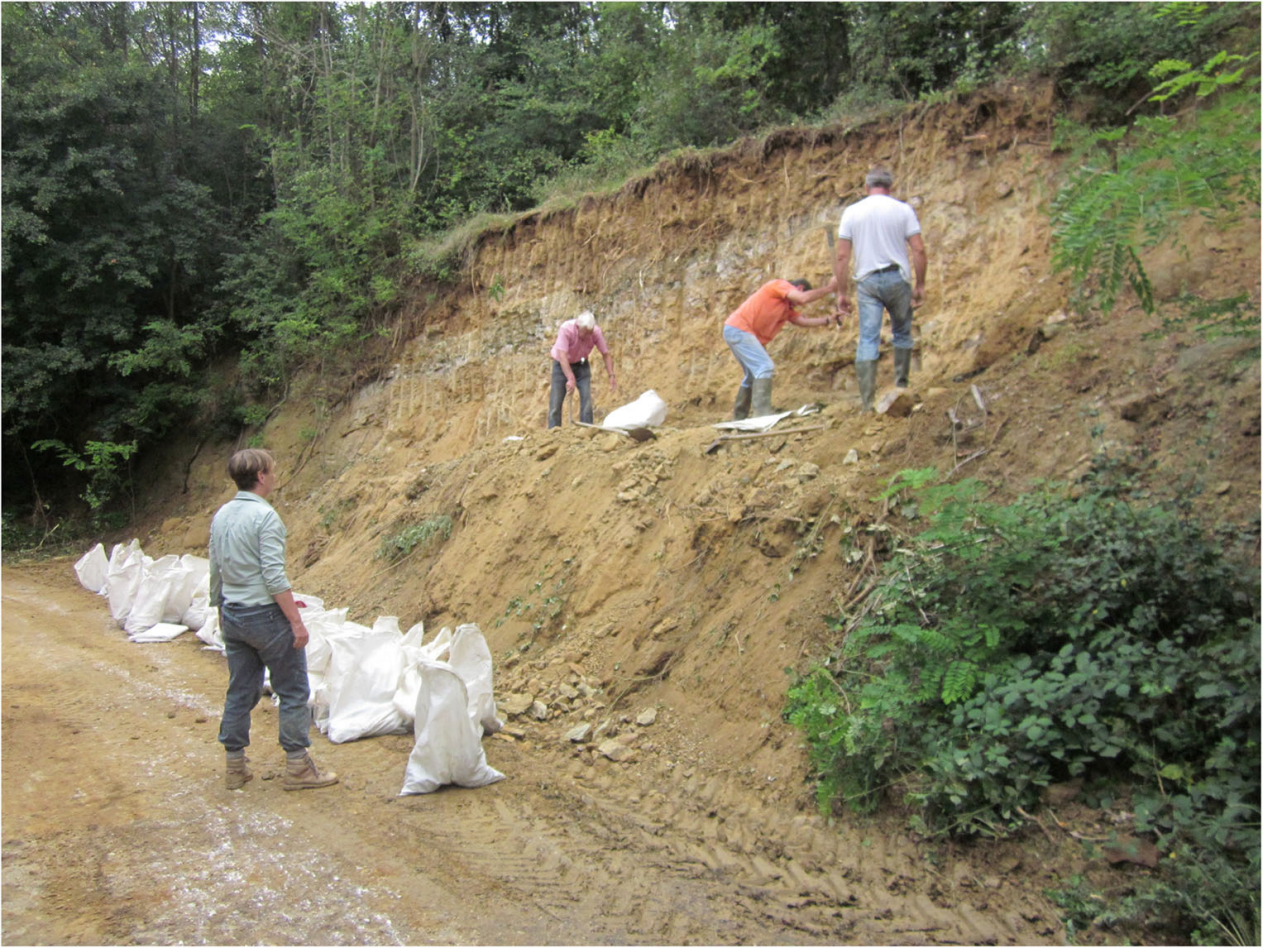
Photograph of the Buštranje road-side locality. White bags are filled with sandy clay from an about 20-cm-thick greenish layer. Over 3 years, about 5000 kg has been taken

For the location of this locality, see Fig. 3; coordinates are 42° 25′ 17″-21° 55′ 48″. The vertebrate remains were collected from a small road-cut outcrop close to a sharp curve about 700 m (as the crow flies) S.E. of the Buštranje church (Figs. 3 and 4). After cleaning, a subhorizontally bedded sequence of carbonate-rich sandstones alternating with more clay-rich sand layers became exposed. The fossil assemblage collected comes from a ~ 20-cm -thick greenish sandy clay.

Based on the mapping by Dimitrijević and Dimitrijević (1981, 1987) the site is near the base of the Sveta Gora Limestone Formation and close to the margin of the area mapped as Buštranje Turbidite Formation (Fig. 2).

The approximate weight of the sample taken is about 5000 kg. Next to small mammal remains, bones of snakes, tortoises and birds have been found; in addition, egg shell fragments and algal balls have been observed in the washed residue.

The fossil content of Buštranje is as follows: Rodentia (see Table 1); Insectivora, Erinaceidae: gen. et sp. indet.; Marsupialia: *Peratherium* sp.

**Table 1:** Distribution table of rodent species in the seven sites of south-east Serbia

			Eocene		Early Oligocene					
Family	Subfamily	Genus and species	Zvonce	Buštranje	Strelac-1	Strelac-2	Strelac-3	Valniš	Raljin	Total M1-M2
Diatomyidae	Diatomyidae	nov. gen. 4 nov. sp.			7	4	3	49	2	65
Dipodidae	Primordial Zapodidae	*Heosminthus borrae*				X	22	20	1	43
Muridae	Pseudocricetodontinae	*Heterocricetodon* nov. sp. A			14	5	6	49	4	78
		*Pseudocricetodon* nov. sp. (small)		29			14			43
		*Pseudocricetodon montalbanensis*			4		23	28	8	63
	Paracricetodontinae	*Paracricetodon dehmi*			3		X	10		13
		*Paracricetodon* nov. sp. B					2	11	?	13
		*Paracricetodon* nov. sp. A		75	45	26	30	127	5	308
	Pappacricetodontinae	*Witenia* sp.			5		X	2		7
		nov. gen.3 nov. sp. A		601						601
		*Witenia* nov. sp. A		21						21
	Melissiodontinae	nov. gen. 2 sp. 2			X			34	1	35
		*Edirnella* nov. sp. 2			6	1				7
		nov. gen. 2 nov. sp. A	28	30						58
		*Edirnella* nov. sp. 1		4		1				5
		cf. *Edirnella* sp. indet	X							1
	?Spalacinae	nov. gen.1 sp. A	3							3
Total number of upper and lower M1 and M2 in each locality			31	760	84	37	100	330	21	1364

Numbers of upper and lower M1 and M2 identified have been indicated; an X indicates that the taxon is not presented by an M1 or M2, but by another dental element

### The Babušnica-Koritnica basin

The basin is located between the towns of Bela Palanka in the northwest and Zvonce in the south-east, close to the border with Bulgaria (Fig. 1). The basin has been mapped as a narrow and elongated NW-SE-oriented basin by the geological survey of Yugoslavia, and details on the basin can be found on map sheets and in the accompanying explanatory notes of sheet Bela Palanka (K_34_33e_R12; Vujisić et al. 1980) and sheet Breznik (K_34_46e_S13; Anđelković and Kristić 1977). Minor parts of the basin are in two other map sheets. Details on the geological structure of the region are in Dimitrijević (1997). Because of proximity to the border, the geology of SW Bulgaria by Zagorchev (2001) is relevant for the understanding the geology. The Babušnica-Koritnica basin is within the tectonic unit of the Carpatho-Balkanides (Fig. 1), which is composed of several long and narrow tectono-stratigraphic units, orientated NNW-SSE that are in fault and/or thrust contact with each other. Strike-slip movements were important at the tectonic boundaries. In the offsetting area of Bulgaria, these boundaries are interpreted as a set of dextral strike-slip faults, named the Struma fault zone (Zagorchev 2001; Fig. 2 in Kounov et al. 2011). The latter defined four Cenozoic tectonic phases: (1) middle Eocene–early Oligocene WSW-ENE extension, formation of grabens and half grabens; (2) intrusion by subvolcanic bodies and dykes during SW-NE extension between 32 and 29 Ma; (3) late Oligocene–earliest Miocene, SSE-NNW transtension and formation of coal-bearing basins; (4) extension since the middle Miocene.

**Fig. 5:**
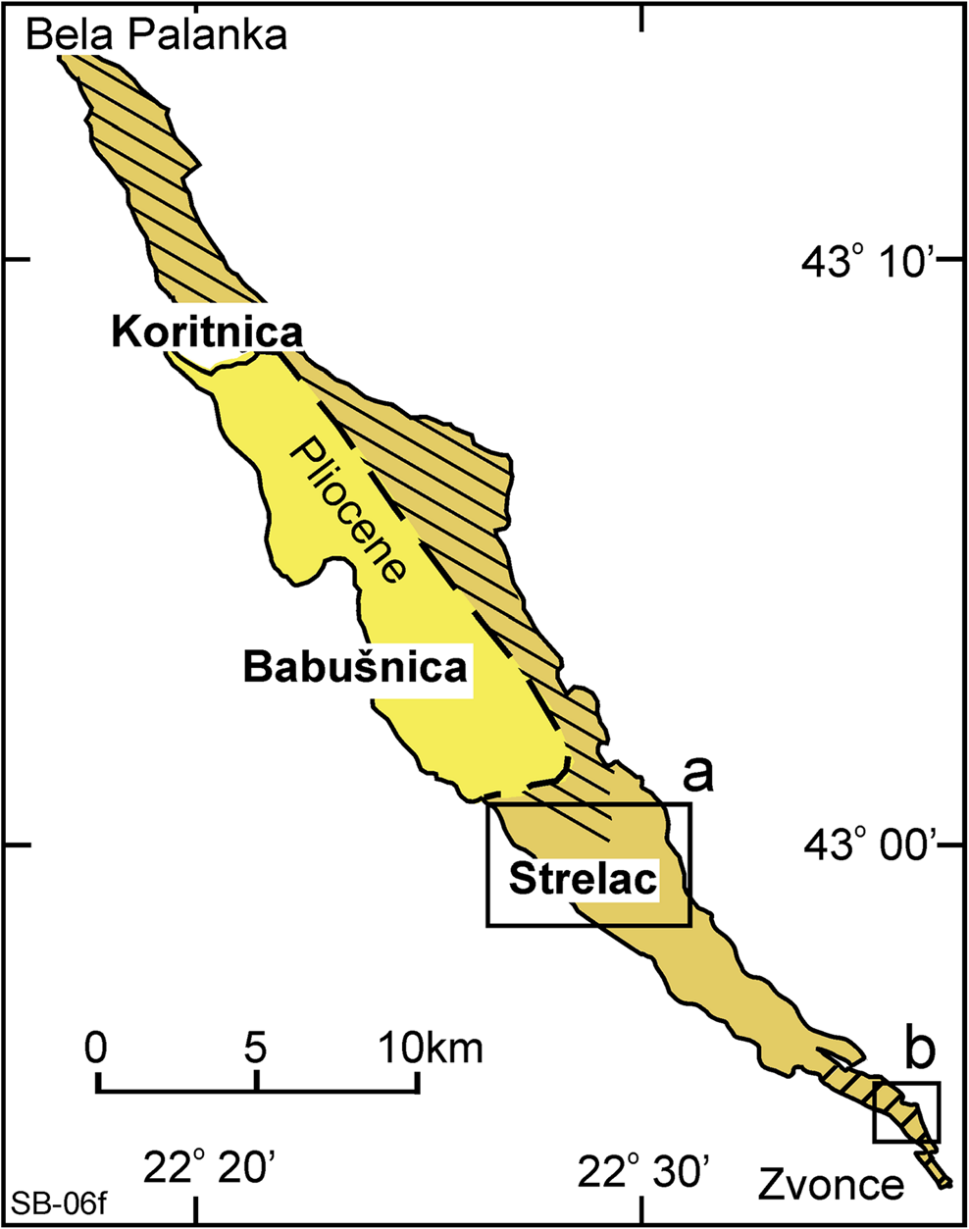
Location map of the fossil localities in the Babušnica-Koritnica basin. The map shows the Paleogene basin fill in brown, the Plio-Pleistocene in yellow. The outline of the basin fill is after the mapping by the geological survey. Three units are provisionally recognised in the Paleogene; see text for further details

The Cenozoic basin fill of the Babušnica-Koritnica basin consists of poorly exposed Paleogene and Plio-Pleistocene clastic sediments (Fig. 5). Detailed studies of the Paleogene in this basin have not been carried out. Below are some observations made during our visits to the basin. From north to south, several depositional units can be recognised (Fig. 5). In the northern half of the basin, a unit of several hundreds of metres thick is present, consisting of thinly laminated siltstones with intercalated volcanic ashes. This unit is deposited in relatively deep lacustrine environments. Fossil fishes (Anđelković 1970) and plant fossils (Mihajlović 1985) have been described from several locations in this unit. This unit is probably of middle–late Eocene Age.

**Fig. 6:**
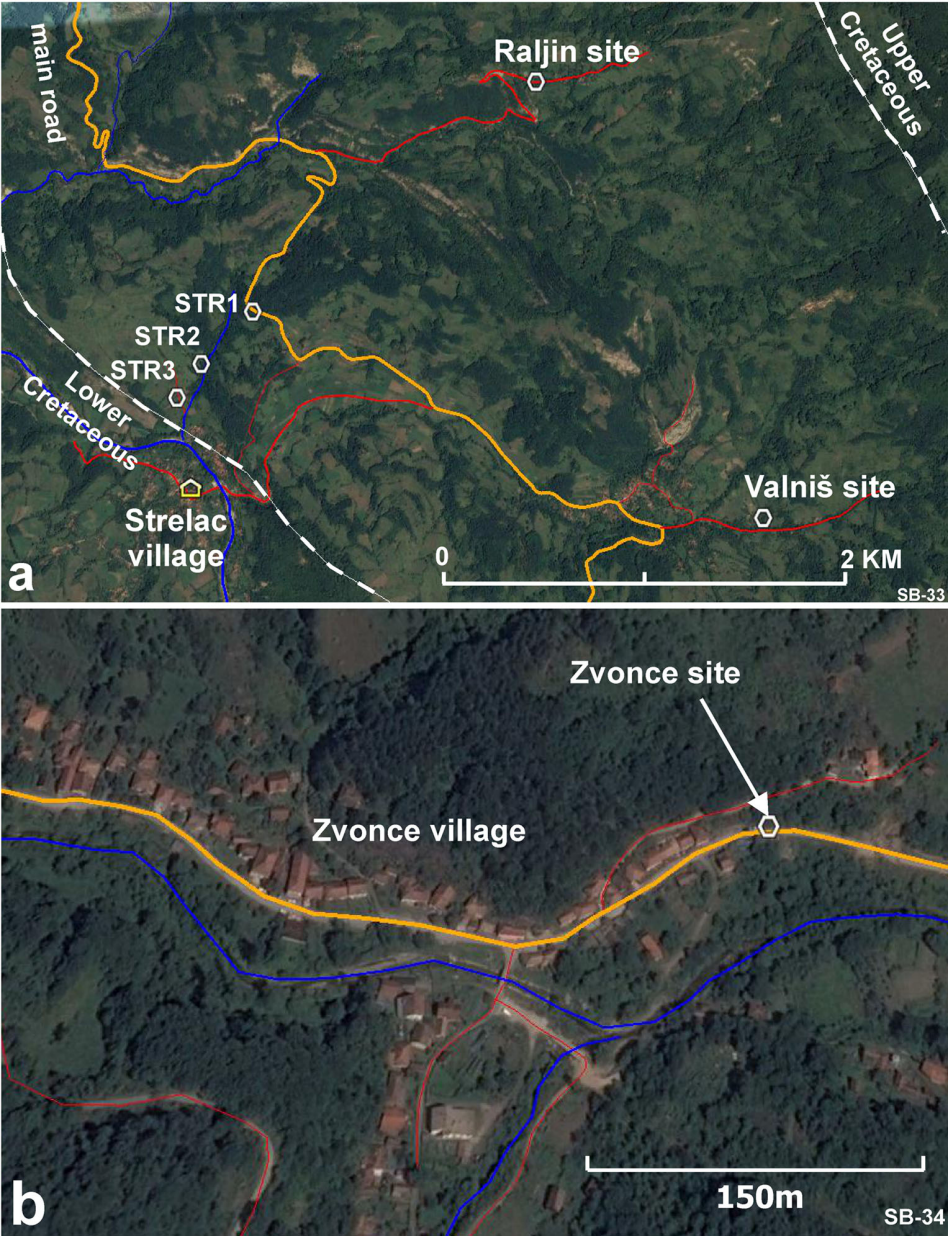
Satellite image of the Strelac area (**a**) and the village of Zvonce (**b**) showing the fossil mammal sites. The areas are indicated on the map of Fig. 5. Main roads are in brown, minor roads in red (satellite images copyrights Google 2017; image CNES/Airbus)

In the area of Strelac are fluvio-lacustrine deposits, sandstones, conglomerates, siltstones and claystones with at places development of coals (Fig. 5). The contact of the fluvio-lacustrine unit with the thinly laminated unit is not exposed, but the contact is probably unconformable, because the thinly laminated unit seems more tectonised. Fossil mammals have been collected from five localities near the village of Strelac: Strelac-1, Strelac-2, Srelac-3, Valniš and Raljin (Fig. 6). Strelac-3 is close to and almost on top of the Lower Cretaceous oolitic limestones (K1 of mapsheet Bela Palanka). At the eastern side of the basin, the Cenozoic is in presumed stratigraphic contact with Upper Cretaceous limestones (K2/3 of mapsheet Bela Palanka). The fossil rodents from this group of five fossiliferous sites suggest an early Oligocene age.

Going from Strelac to the south, the fluvio-lacustrine unit tends to change colour to reddish hues and shows an increase of coarse intercalations. Near Zvonce, Cenozoic rocks have been mapped as a narrow strip in faulted contact with Upper Jurassic and Lower Triassic carbonates (Anđelković et al. 1968, mapsheet Breznik). The area is strongly tectonised with steep dips. The Cenozoic in this location consists of a succession of indurated clays and sandstones. The sandstones are well-bedded and suggest being deposited in limnic turbiditic facies, possibly close to a fluvial delta. The collected fossil rodents from Zvonce suggests an Eocene age.

In the middle part of the basin, a lithological unit of sands and clays is present that is unconformable on the thinly laminated Eocene unit (Fig. 5). This unit is probably Plio-Pleistocene in age, but fossils allowing precise dating have not been found. The differences in age and depositional facies and the tectonic complexity suggest a multi-phase origin for the Babušnica-Koritnica basin.

Fossil small mammals have been collected in six localities in this basin: one locality in the village of Zvonce, three localities near the village of Strelac, one locality near Valniš and one locality near the hamlet of Raljin.

#### The fossil mammal locality Zvonce

**Fig. 7:**
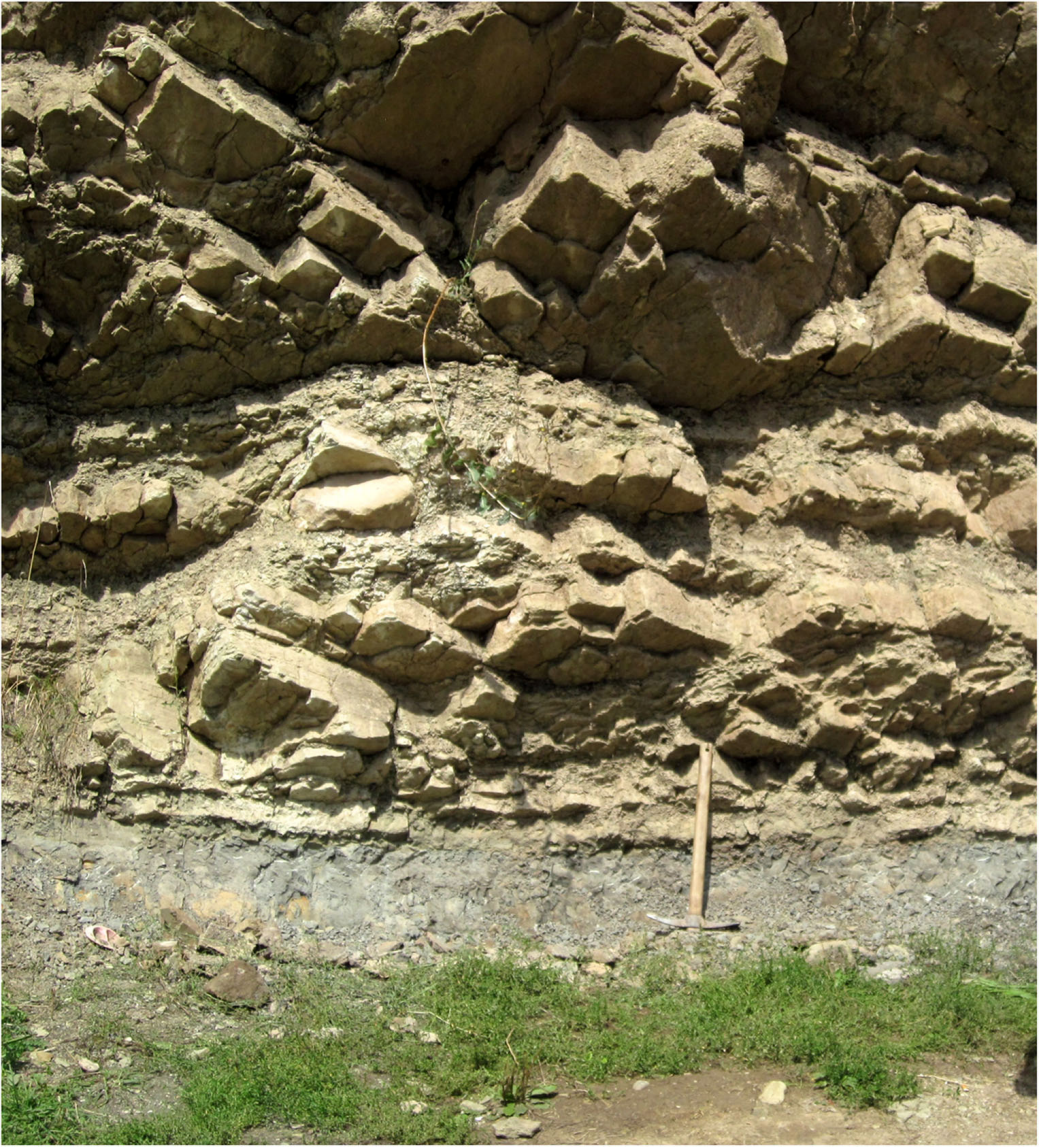
The Zvonce locality. The sampled clay is about 30 cm thick and located just at street level. It is overlain by an about 3-m-thick steeply dipping well-bedded sandstone. Hammer for scale

For the location of this site (Fig. 6), coordinates are 42° 55′ 54″-22° 34′ 43″. The locality Zvonce is situated in a road-side outcrop of possibly proximal turbiditic deposits next to the grocery shop annex cafe in the village of Zvonce. The fossiliferous bed is a dark-grey indurated clay just above street level (Fig. 7). The approximate weight of the sample taken in this location is about 1000 kg.

The fossil content of Zvonce is as follows: Rodentia (see Table 1); Insectivora indet., Artiodactyla indet., Carnivora indet. In addition, casts and operculae of gastropods have been found.

#### The fossil mammal locality Strelac-1

**Fig. 8:**
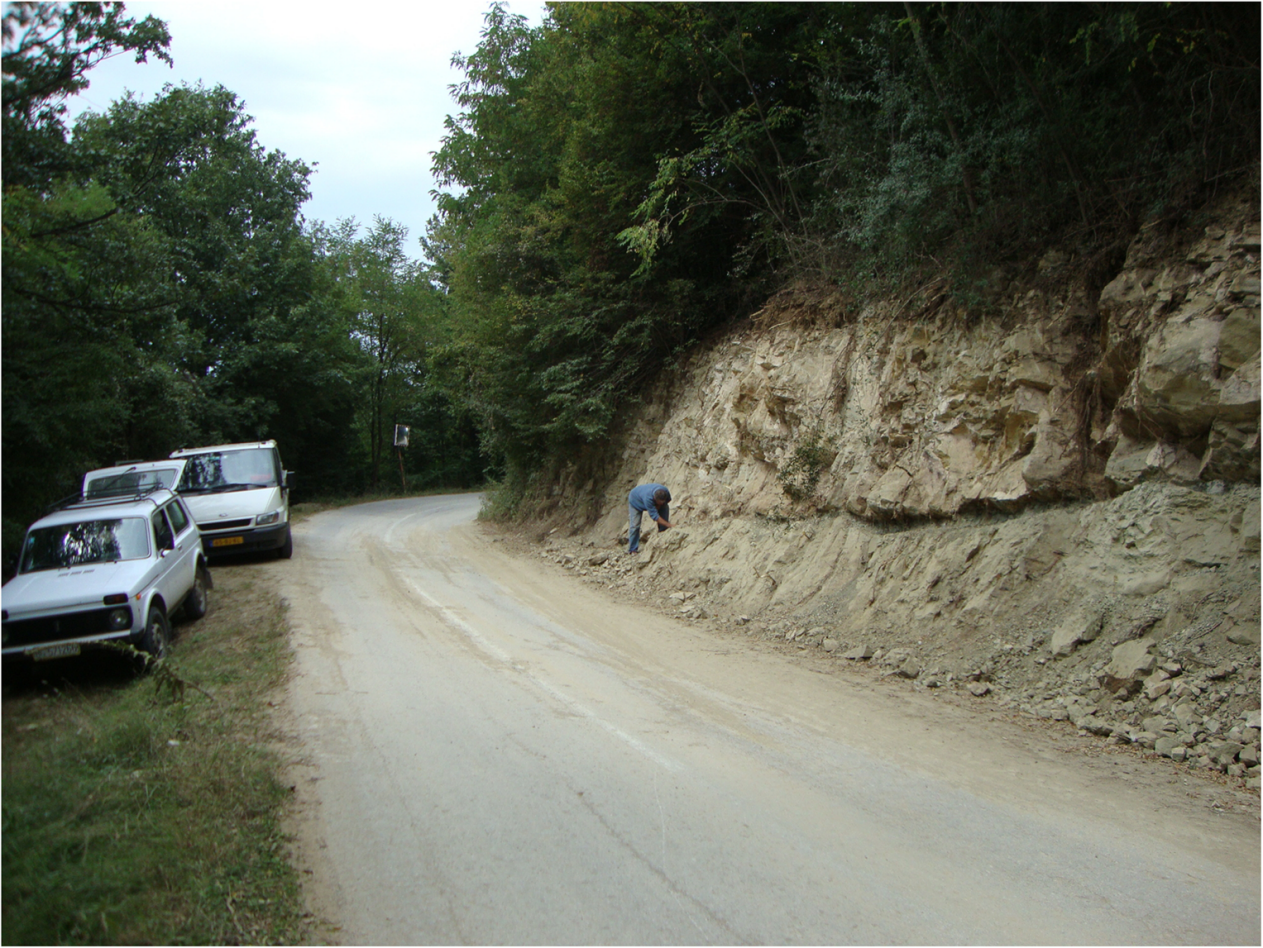
The Strelac-1 locality along the road between Babušnica and Zvonce. The sampled clay is about 15 cm thick and located below a thick sandstone

For the location of this locality (Fig. 6), coordinates are 42° 59′ 36″-22° 28′ 03″. The sampled locality is in an about 4-m-high outcrop on the north side of the road from Babušnica to Zvonce directly above the village of Strelac (Fig. 8). The fossils originate from a greenish clay layer of irregular thickness (~ 15 cm thick) with some coal fragments. The fossiliferous clay layer is overlain by several metres-thick very hard coarse sandstones which could not be moved by means of a digging machine. The approximate weight of the sample taken is about 1500 kg.

The fossil content of Strelac-1 is as follows: Rodentia (see Table 1); Insectivora: Erinaceidae gen. et sp. indet.; Artiodactyla: gen. et sp. indet; freshwater molluscs.

#### The fossil mammal locality Strelac-2

For the location of this locality (Fig. 6), coordinates are 42° 59′ 25″-22° 27′ 53″. The locality Strelac-2 is situated along the path north-east of the village of Strelac leading uphill into the forest and some 20 m beyond where this path crosses a brook. The sedimentary sequence exposed is similar as in Strelac-1, but the overlying sandstone is much thinner. The clay layer from which the fossils were collected is irregular in thickness (~ 20 cm) and more bluish/reddish. In this layer, coal inclusions, sand grains of different sizes and small pebbles have been observed. The approximate weight of the sample taken is about 900 kg.

The fossil content of Strelac-2 is as follows: Rodentia (see Table 1); Insectivora: Erinaceidae gen. et sp. indet.; Artiodactyla: gen. et sp. indet.; Marsupialia: *Peratherium* sp.; freshwater molluscs.

#### The fossil mammal locality Strelac-3

For the location of this site, see Fig. 6; coordinates are 42° 59′ 20″-22° 27′ 47″. The locality Strelac-3 is situated along the old hollow-road parallel to the river north-east of the village of Strelac. The small outcrops on both sides of that road show whitish carbonate-rich marls and sandy clay layers with some coal fragments. Stratigraphically, this outcrop is close to the underlying Cretaceous limestones, but the contact is not exposed. The approximate weight of the sample taken is about 1500 kg.

The fossil content of Strelac-3 is as follows: Rodentia (see Table 1); Artiodactyla: gen. et sp. indet.; Marsupialia: *Peratherium* sp.; other vertebrates: crocodile, lizard, tortoise and frog remains. Egg shell fragments, in addition, charophyte oögonia, gastropods and gastroliths have been observed.

#### The fossil mammal locality Valniš

For location of this locality (Fig. 6), coordinates are 42° 59′ 02″-22° 29′ 59″. The locality Valniš is situated in the west bank of the hollow path leading uphill east of the village of Valniš next to a collapsed gallery of a deserted mine of low-grade coal. The fossils are from a 100–150-cm-thick coal-rich clay layer with abundant mollusc remains which is overlain by a thick bed of clean whitish unconsolidated sand. Although fossils occur throughout the layer, the slightly indurated top of about 20 cm appeared to be the most productive. The approximate weight of the sample taken is about 4500 kg.

The fossil content of Valniš is as follows: Rodentia (see Table 1); Insectivora: Erinaceidae: gen. et sp. indet.; Marsupialia: *Peratherium* sp.; other vertebrates: crocodile, lizard, tortoise and frog-remains. Egg shell fragments, charophyte oögonia, poorly preserved seeds, gastropods and gastroliths have been observed.

#### The fossil mammal locality Raljin

For the location of this site (Fig. 6), coordinates are 43° 00′ 14″-22° 29′ 06″. The locality Raljin is situated in the bank of the unpaved road of the village. The circa 3-m-high outcrop shows a sequence of sandy marls on top of a 75-cm-thick clay-coal interval rich in mollusc remains and gypsum crystals. The stratigraphical position of the fossiliferous deposit is almost directly on top of the Eocene thinly laminated unit, but the contact is not exposed. The approximate weight of the sample taken is about 2000 kg.

The fossil content of Raljin is as follows: Rodentia (see Table 1). Insectivora: Erinaceidae: gen. et sp. indet.; Marsupialia: *Peratherium* sp.; Carnivora indet.; other vertebrates: crocodile, snake, lizard, frog and tortoise-remains. Invertebrates and plants: charophyte oögonia, poorly preserved micro-flora, gastropods and gastroliths.

## The rodent assemblages

### Introduction

The collection of Paleogene rodents from the localities in south-east Serbia described in this paper contains to date about 1500 isolated cheek teeth of the families Diatomyidae, Dipodidae and Muridae. Rodent taxa represented by one specimen will be omitted in this overview. The Muridae are, with five subfamilies and eight genera, by far the most diverse and abundant group (Table 1) and dominate all seven fossil assemblages discussed.

The allocation of primordial cricetid genera to a specific subfamily is often problematic; some of these have been shown to be clades, i.e. the Melissodontinae, Pseudocricetodontinae and Paracricetodontinae (Kalthoff 2006), while others, such as the Pappocricetodontinae, containing an array of genera with very large as well as very small species, which share primitive dental characteristics only, are obviously polyphyletic. Tracing the roots of a particular well-defined subfamily from the Oligocene of Europe among Eocene Asian material is problematic and has resulted in very different conclusions (Maridet and Ni 2013; Gomes Rodrigues et al. 2009). The assemblages of Zvonce and Buštranje from the (late?) Eocene of respectively the Babušnica-Koritnica and Pčinja basins differ sharply from each other as well as from the early Oligocene ones (Tables 1 and 2). The composition and content of the five, presumably early Oligocene, rodent assemblages from the Strelac area in Babušnica-Koritnica basin are very similar on the species level, so these are considered to constitute one “Local Fauna”.

**Table 2:** Comparison between the Eocene sites (Zvonce and Buštranje) and the Oligocene sites (Strelac-1, Strelac-2, Strelac-3, Valniš and Raljin)

Family	Subfamily/genus		Eocene	Oligocene
Diatomyidae	new genus		0	65
Dipodidae	*Heosminthus*		0	43
Muridae	Pseudocricetontinae	*Heterocricetodon*	0	78
		*Pseudocricetodon*	29	77
	Paracricetodontinae		75	259
	Pappacricetodontinae		622	7
	Melissiodontinae		63	43
	?Spalacinae		3	0
Total number of upper and lower M1 and M2			792	572

### Remarks on the rodent (sub)families

#### Diatomyidae Mein and Ginsburg, 1997

**Fig. 9:**
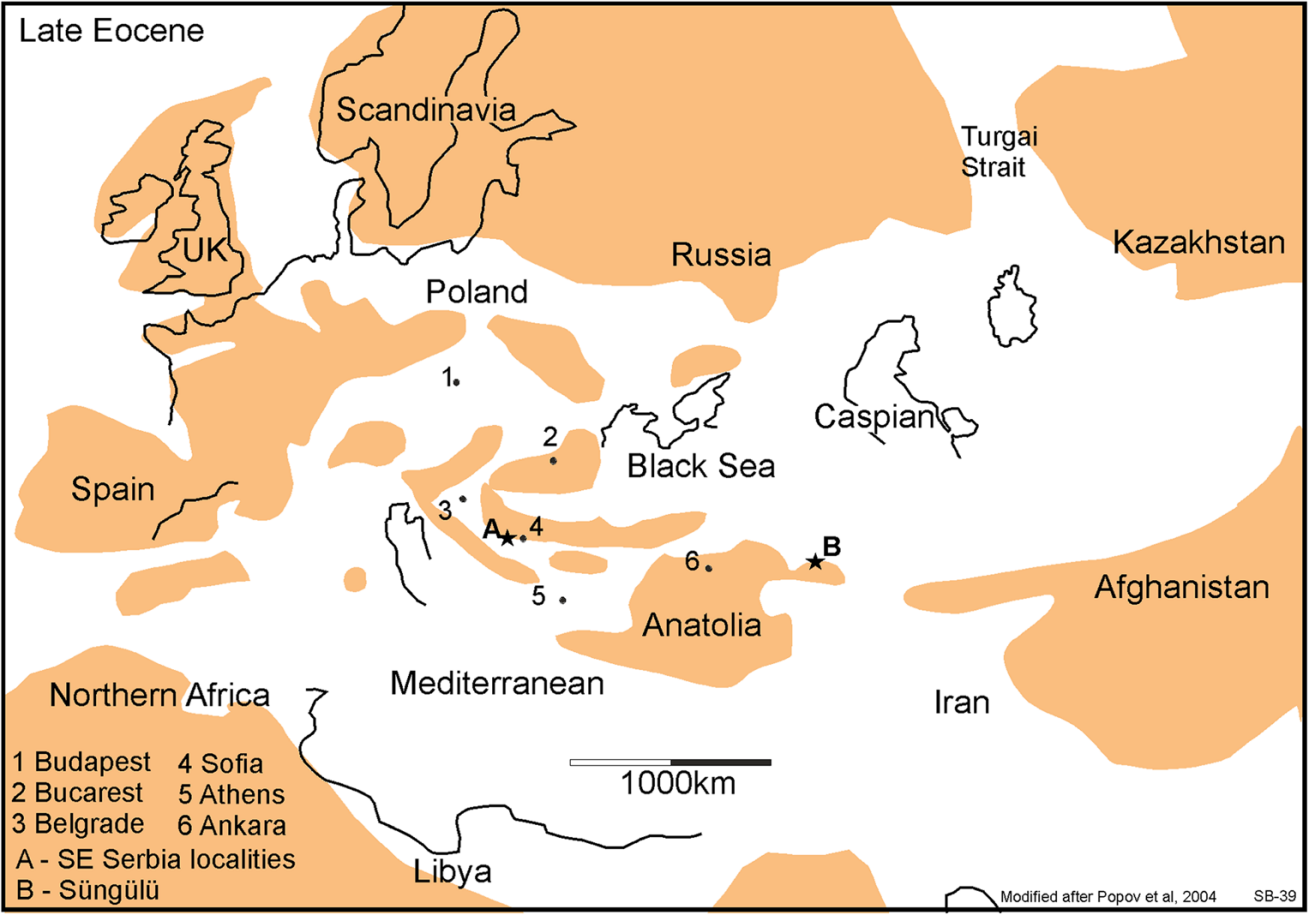
Palaeogeographic sketch of Europe, Northern Africa and the Middle East showing the distribution of land areas (brown) and sea (white) during the late Eocene. For ease of reference, the approximate position of some present-day capital cities have been indicated. The location of the south-east Serbian localities with fossil rodents is west of Sofia and indicated with star A, the late Eocene site of Süngülü is indicated by star B. The sketch shows the fragmentation of land areas between Anatolia and western Europe. The sketch is strongly simplified after Popov et al. (2004)

**Fig. 10:**
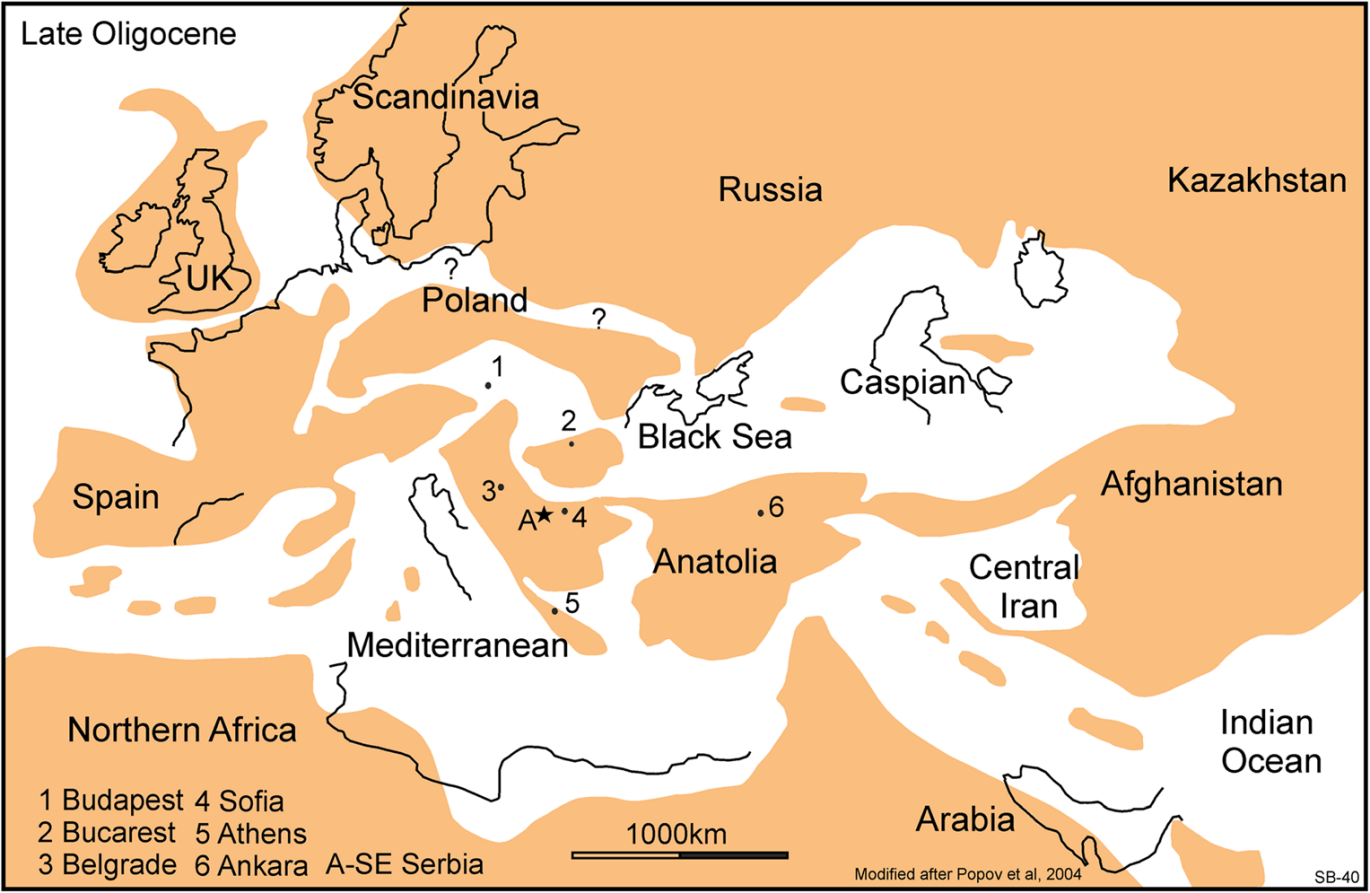
Palaeogeographic sketch of Europe, Northern Africa and Middle East showing the distribution of land areas (brown) and sea (white) during the late Oligocene. The potential land migration routes through Afghanistan, Anatolia, the Balkan and Western Europe have strengthened resulting in an isolation of the Paratethys. The sketch map is strongly simplified after Popov et al. (2004)

The family Diatomyidae has so far been considered to be restricted to central and south-east Asia throughout their history (Flynn et al. 1986; Marivaux et al. 1999), so its presence in Serbia is surprising. The five localities near Strelac share the same species of diatomyid. Its teeth are similar to, but more primitive than, those of *Fallomus razae* Flynn, Jacobs and Cheema, 1986. In the Serbian material, the third molars are larger than the second molars, the D4 has an endoloph and the lower molars have an ectolophid as well as a prominent hypoconulid. *Fallomus razae* from the Oligocene of Baluchistan and western Pakistan (Flynn et al. 1986; Marivaux and Welcomme 2003; Flynn 2007) is so far the oldest representative of the family. The presence of a primitive diatomyid in the early Oligocene of Serbia suggests that the area of their origin may not have been the Indian subcontinent as is generally assumed, but rather somewhere in the eastern part of the Paratethys, which was littered with islands during the Paleogene (Figs. 9 and 10; Meulenkamp and Sissingh 2003; Popov et al. 2004). The original idea of Flynn et al. (1986) that the Diatomyidae evolved from a primitive isolated ctenodactyloid stock still remains the most probable scenario.

#### *Dipodidae Fischer von Waldheim, 1817*

**Fig. 11:**
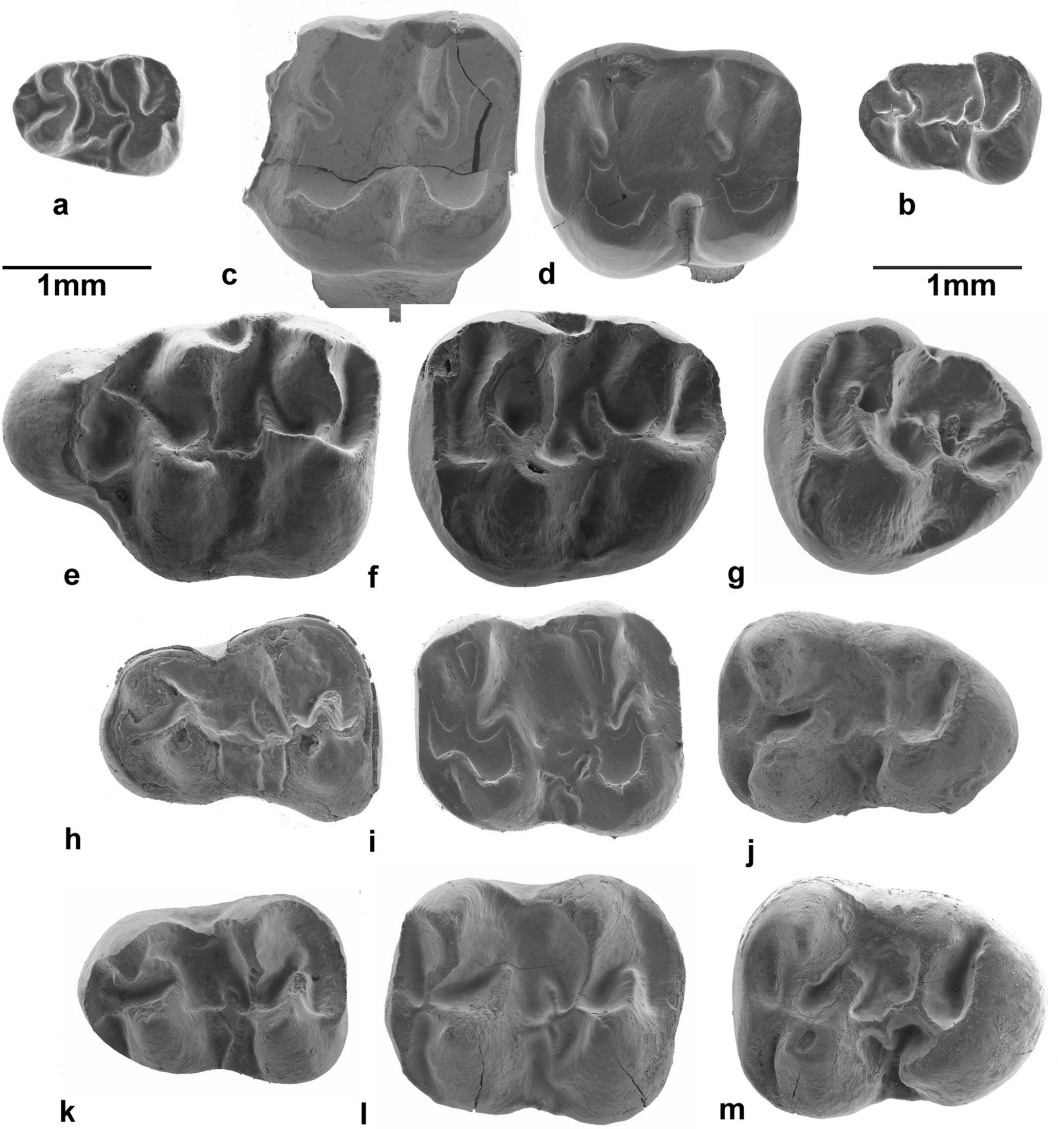
*Paracricetodon dehmi* from Valniš and Strelac compared with *Paracricetodon dehmi* from Belgarite (France). The m1 of *Heosminthus borrae* and *Pseudocricetodon montalbanensis* are added to show the size differences. *Heosminthus borrae*: **a**: m1 (STR3-145). *Pseudocricetodon montalbanensis*: **b**: m1 (VA-438). *Paracricetodon dehmi*: from Valniš and Strelac: **c**: M1 (STR1-281); **d**: M2 (VA-856); **h**: m1 (VA-862); **i**: m2 (VA-866); **j**: m3 (STR1-288). *Paracricetodon dehm*i from Belgarite: **e**: M1 (BEL-401); **f**: M2 (BEL-403); **g**: M3 (BEL-404); k m1 (BEL-403); **l**: m2 (BEL-406); **m**: m3 (BEL-407)

**Fig. 12:**
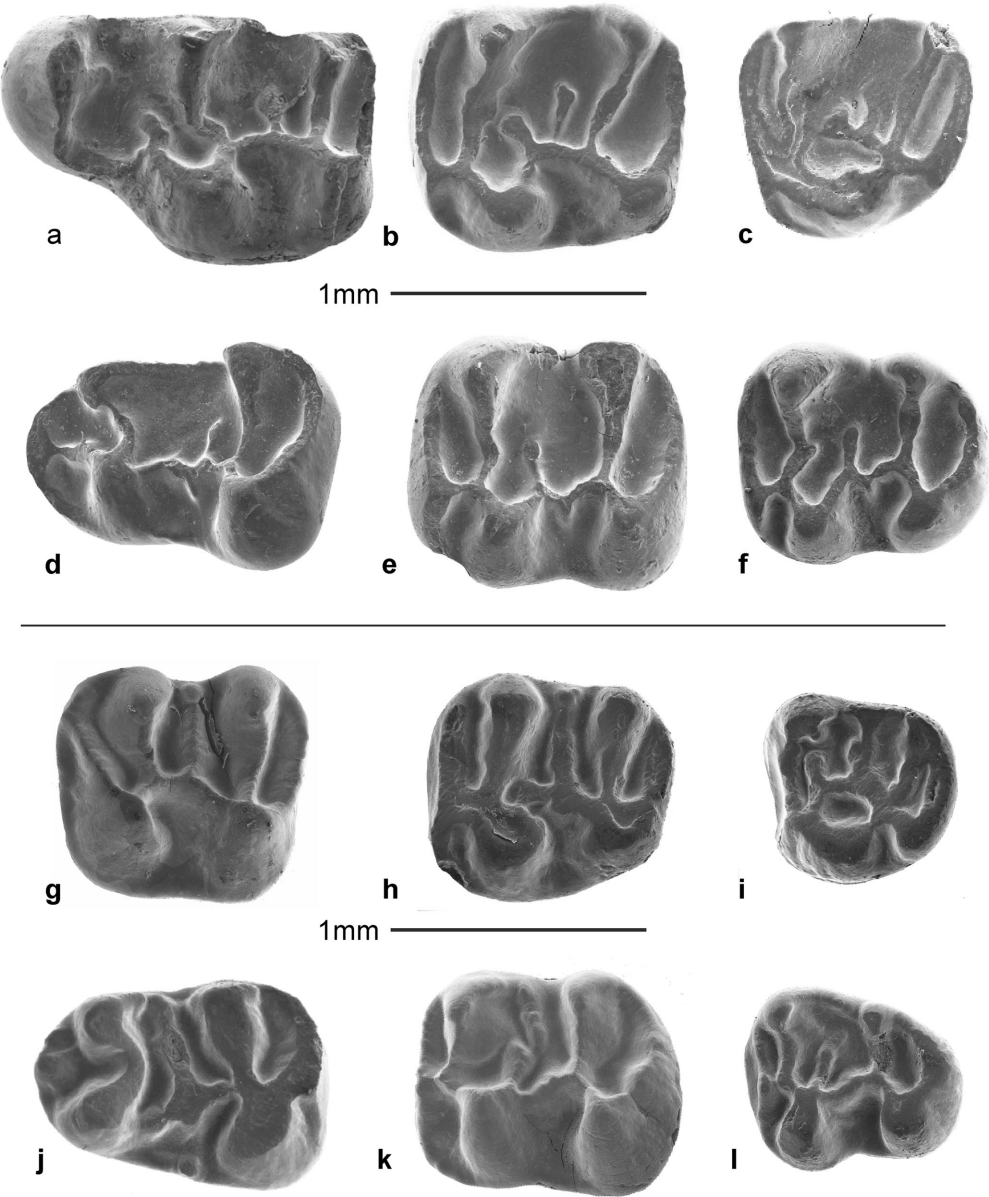
*Pseudocricetodon montalbanensis*. **a**: M1 (STR1-131); **b**: M2 (VA-416); **c**: M3 (VA-422); **d**: m1 (VA-438); **e**: m2 (VA-442); **f**: m3sin (VA-452). *Heosminthus borrae*: **g**: M1 (STR3-117); **h**: M2 (STR3-127); **i**: M3 (STR3-135); **j**: m1 (STR3-145); **k**: m2 (STR3-342); **l**: m3 (STR3-165)

The extant members of the family Dipodidae occupy a wide range of ecological niches and are morphologically most heterogeneous. Since none of the highly specialised extant adaptive types can be traced back beyond the Miocene, allocation to subfamily of the primordial Dipodidae genera remains unsettled. While McKenna and Bell (1997) synonymise most Asian Paleogene genera known at the time—*Parasminthus* Bohlin, 1946, *Sinosminthus* Wang, 1985, *Heosminthus* Wang, 1985, *Gobiosminthus* Huang, 1992, *Shamosminthus* Huang, 1992—with *Plesiosminthus* Viret, 1926. Others, like Tong (1997), Lopatin (1999) Daxner-Höck (2001) and Daxner-Höck et al. (2014) defined new genera on the basis of minor dental differences. Judging by the literature, neither approach has been leading to a satisfactory scheme, so revision of the Asian Paleogene record of the group seems badly needed. The dipodid material from the Oligocene of south-east Serbia is tentatively assigned to the genus *Heosminthus* Wang, 1985 (possible synonyms are *Bohlinosminthus* Lopatin, 1999 and *Tatalsminthus* Daxner-Höck, 2001), and the species *Heosminthus borrae* Daxner-Höck, 2014 (Figs. 11 and 12). Although the upper incisor of our small dipodid could not be identified, we assume that it had a flat anterior surface because none of the incisors from Strelac-3 and Valniš, the two sites where it is represented best, has a sulcus. M1 and M2 have three roots; the metaloph of the M2 inserts on the hypocone or on the anterior arm of the hypocone and the mesolophs(ids) of the first and second molars are rather long. *Heosminthus borrae* occurs in Strelac-2 and Strelac-3, Valniš and Raljin (Table 1).

### Muridae Illiger, 1811

#### Pseudocricetodontinae Engesser, 1987

The cheek teeth of the type species of *Pseudocricetodon* (*P. montalbanensis* Thaler, 1969) and *Heterocricetodon* (*H. stehlini* Schaub, 1925) are quite different. In spite of this, Engesser (1987) united these two genera into his subfamily Pseudocricetodontinae. This action, though contested by Kristkoitz (1992) on the basis of differences in skull characteristics, was strongly supported by the study of the incisor enamel microstructure (Kalthoff 2006) and is now generally accepted. However, the contents of this Pseudocricetodontinae, in particular the incorporation of the genus *Adelomyarion*, as suggested by Kalthoff (op. cit.) has been disputed (Freudenthal et al. 1992). An additional problem is that the incisor enamel of a number of genera that are potentially members of the subfamily has not yet been studied. Our working hypothesis is that the subfamily Pseudocricetodontinae includes the genera *Pseudocricetodon* Thaler, 1969 (? = *Allocricetodon* Freudenthal, 1994, pro parte), *Heterocricetodon* Schaub, 1925 (? = *Allocricetodon* Freudenthal 1994, pro parte), *Adelomyarion* Hugueney, 1969, *Lignitella* Ünay-Bayraktar, 1989, *Kerosinia* Ünay-Bayraktar, 1989, *Cincamyarion* Agusti and Arbiol, 1989, *Eocricetodon* Wang, 2007 (? = *Pseudocricetodon*) and *Oxinocricetodon* Wang, 2007 (? = *Heterocricetodon*). If so understood, the subfamily has a Eurasian range from the (late?) Eocene to the early Miocene.

*Pseudocricetodon* is present in all our Paleogene Serbian assemblages except Zvonce and Strelac-2 (Table 1). If our age allocations are correct, the species from Buštranje (smaller than *Pseudocricetodon montalbanensis*) are the oldest occurrence anywhere. The assemblages from the Strelac area all contain *P. montalbanensis* (Table 1; Figs. 11 and 12)*.* In addition to this species, which has an exceptionally wide stratigraphical and geographical range, there is a smaller species in the collections from Buštranje and Strelac-3. An M3 and m2 of an enigmatic lophodont cricetid with a very complex dental pattern from Valniš is tentatively listed as cf. Pseudocricetodontinae nov. gen. nov. sp.

*Heterocricetodon* nov. sp. A. is represented in all five localities in the Strelac area by a medium-sized species, with low-crowned, lophodont elongate cheek teeth. Like the type species *Heterocricetodon stehlini*, the three lower cheek teeth have about the same length, while the M2 are on average longer than wide and the length and width of the M3 are approximately equal.

#### Paracricetodontinae Mein and Freudenthal, 1971

We restrict the content of the subfamily Paracricetodontinae to *Paracricetodon* Schaub, 1925, and *Trakymys* Ünay-Bayraktar, 1989, that is to genera with species having lower incisor enamel with a type 8 Schmelzmuster (Kalthoff 2000). This is not only in sharp contrast to the classification by McKenna and Bell (1997), who include the majority of the European as well as some North American Paleogene cricetids into this subfamily, but also deviates from the classifications suggested by Freudenthal et al. (1992) and Kalthoff (2006), in excluding *Edirnella* Ünay-Bayraktar, 1989. New evidence has shown that the type species of *Edirnella, E. sinani,* has a type 1 microstructure in the lower incisor, which characterises the Melissodontinae.

*Paracricetodon* is present in all our Paleogene assemblages except the one from Zvonce (Table 1). Three species are recognised: the smallest of these, *Paracricetodon* nov. sp. A, is rather common in Buštranje as well as in the localities in the Strelac area. Its cheek teeth are morphologically quite similar to *Paracricetodon wentgesi* de Bruijn et al. 2003, from the Eo-Oligocene boundary interval (Süngülü, Lesser Caucasus; for location, see Fig. 9, star B). The teeth of *Paracricetodon* nov. sp. A are somewhat smaller than the ones of *P. wentgesi*, but there is considerable overlap in size. The main difference between these species is in the degree of reduction of the M3/m3. This is so much so that the most elaborate M3/m3 from Serbia is more simply built than the most reduced specimen from Süngülü. The specimens from Buštranje are on average somewhat smaller than the ones from Strelac-1, Strelac-2, Strelac-3, Valniš and Raljin. The second smallest species (*Paracricetodon* nov. sp. B) has relatively narrow teeth, is rare and occurs in Valniš and Strelac-3 only. The largest of the three *Paracricetodon* species recognised, allocated to *Paracricetodon dehmi* Hrubesch, 1957, is present with a few isolated teeth in the assemblages from Valniš and Strelac-1. These are illustrated in Fig. 11 together with a few specimens of the same species from the Oligocene site of Belgarite (France) for comparison.

#### Pappocricetodontinae Tong, 1997

The subfamily Pappocricetodontinae, type *Pappocricetodon rencunensis* Tong, 1992, was created to house the small middle Eocene cricetids *Pappocricetodon* Tong, 1992, and *Palasiomys* Tong, 1997. A third primitive cricetid of Eocene age, *Raricricetodon* Tong, 1997—originally the type of the subfamily Raricricetodontinae Tong, 1997—was later included in the Pappocricetodontinae. Since these genera are very close to the divergence of the Muridae from the Dipodidae, allocation to family of taxa that are exclusively known by isolated cheek teeth is problematic. Although the information on the Eocene and Early Oligocene Muridae has been rapidly growing during the last two decades, some of the genera defined, such as the small *Ulaancricetodon badamae* Daxner-Höck, 2000, were not allocated to the subfamily level by their authors, while others, such as the large *Witenia* species, were included in the Pappocricetodontinae (de Bruijn et al. 2003), an action that devaluated the subfamily into a grade. The association from Buštranje contains two Pappocricetodontinae candidates: (1) a very abundant small species with a rather complex dental pattern that shows a surprisingly wide range of morphological variation and (2) a relatively rare very large species with teeth that show an extremely primitive morphology. The first is listed as Pappocricetodontinae nov. gen. A, nov. sp. 1, the second as *Witenia* nov. sp. 1. The early Oligocene sites Strelac-1 and Valniš yielded a somewhat smaller and more derived species of *Witenia* that is listed as *Witenia* nov. sp. 2.

#### Melissiodontinae Schaub, 1925

The species assembled in the traditionally monogeneric subfamily Melissiodontinae share a set of dental, cranial and mandibular characteristics (Kristkoiz 1992) that distinguishes them from all other Muridae. This so much so that, although Ünay-Bayraktar (1989) and de Bruijn et al. (2003) suggested more diversity by including *Edirnella* into the subfamily, its content remained restricted to *Melissiodon* Schaub, 1925, in most classifications (Freudenthal et al. 1992; Kalthoff 2006). The presence of an array of different melissiodontines in the Paleogene assemblages from Serbia is therefore a surprise. We include three genera: *Melissiodon*, *Edirnella* and a new genus. Our decision to include *Edirnella* in the subfamily is based on new evidence of the enamel structure of the lower incisor from the type species.

The presumably Eocene assemblages from Zvonce and Buštranje each contain a species of *Edirnella* next to a small, low-crowned member of our new genus, while a second, slightly larger and dentally much more complex species of this genus occurs associated with a somewhat smaller species of *Edirnella* in some of the localities of the Early Oligocene near Strelac (Table 1). The Serbian record of the melissiodontines shows that this peculiar branch of the Muridae, combining highly derived cheek teeth with the primitive type 1 schmelzmuster (Kalthoff 2006) in the lower incisor, is much older than previously assumed and seems to have originated by isolation in the Eocene forest (Mihajlović 1985) of the Serbo-Macedonian land area.

The weak long mandible with a shallow masseter scar is very similar to that of the shrew rats of the Philippines and Sulawesi (Hordijk et al. 2015), so a similar insectivorous diet is assumed for the melissiodontines.

## The composition of the rodent assemblages

### Eocene

The rodent associations from Zvonce and Buštranje, different though they are, contain Muridae only (Tables 1 and 2). Buštranje has species with very derived as well as very primitive dentitions. The presence of a melissiodontine and what seems to be a spalacine in Zvonce and of the combination of pappocricetodontines and melissiodontines with *Pseudocricetodon* and *Paracricetodon* in Buštranje is so far unique. Remarkable is that *Eucricetodon* Thaler, 1966, the first cricetid to arrive in central and southwestern Europe after the “Grande Coupure”, is absent in this community. This is also the case for the ctenodactyloids, which dominate the Paleogene rodent assemblages of Asia.

### Oligocene

The rodent associations from Strelac-1, Strelac-2, Strelac-3, Valniš and Raljin contain a dipodid and a diatomyid species, but the Muridae remain the dominating family (Tables 1 and 2). Both these species as well as *Heterocricetodon* seem to be immigrants. The rare pappocricetodontines are represented in these assemblages by a derived *Witenia*, while *Eucricetodon* remains absent. The Ctenodactylidae are represented in the assemblage of Valniš by one P4 and a damaged m3 only.

## The age of the rodent assemblages

Since our fossil localities are neither situated in well-exposed long sections nor associated with primary volcanic rocks, the dating of these associations is exclusively based on biostratigraphy. However, straightforward correlation of the murid-dominated Paleogene rodent association of Serbia with the theridomyid-dominated succession of southwestern Europe or the ctenodactylid-dominated succession of Central Asia is hampered by the paucity of taxa shared. Other than the prudent age estimates by Mihajlović (1985) based on macroflora, we depend on comparison of the stage of evolution of rodent dentitions with elements from different fauna successions in different provinces. The age assignments therefore have necessarily a relatively wide range. The assemblages from Zvonce and Buštranje are considered to be roughly coeval because they share the melissiodontine (listed as nov. gen. 2, nov. sp. A, Table 1). Their (?late) Eocene age is based on the stage of evolution of this species, which is much more primitive than the oldest central European melissiodontine, *Melissiodon bernlochense* Hrubesch, 1957, from the locality Bernloch (MP23; Germany), as well as on the presence in Buštranje of a large species of *Witenia* with cheek teeth that are morphologically very similar to those of *Pappocricetodon antiquus* Wang and Dawson, 1994, from the middle Eocene of China. The Buštranje rodent fauna shows some affinity with the one from Süngülü (de Bruijn et al. 2003; Lesser Caucasus, see Fig. 9) in containing the genera *Edirnella*, *Pseudocricetodon*, *Paracricetodon* and *Witenia*, though these are represented by different species in Serbia.

Our (?late) Eocene age attribution of the Buštranje rodent fauna in the Pčinja basin is similar to the Priabonian age given by Dimitrijević and Dimitrijević (1981, 1987) to the formation from which our fossil rodents have been collected. However, reasons for this age assignment were not presented by these authors.

The assignment to the Oligocene of the localities in the Strelac area is based on the presence of *Heosminthus borrae* originally described from the early Oligocene of Mongolia (Daxner-Höck et al. 2014), *Pseudocricetodon montalbanensis* from the early Oligocene (MP23, Spain; Thaler 1969) and *Paracricetodon dehmi* Hrubesch, 1957, from the early Oligocene (MP23 of Germany; Hrubesch 1957). Further, evidence supporting the early Oligocene age of these associations is provided by the stage of evolution of the diatomyid, which is similar to, but more primitive than, the dentition of *Fallomus razae* from the Oligocene of Baluchistan (Flynn et al. 1986). The associations from the Strelac area show some affinity with the one from Paali Nala (Baluchistan; Marivaux et al. 1999) in sharing the presence of a primitive diatomyid and *Pseudocricetodon.*

## Palaeogeography and migrations

The Eocene-Oligocene transition is marked by a rapid cooling of the climate. Based on detailed studies in the Hampshire and Ebro basins (Köhler and Moyà-Solà 1999; Costa et al. 2011; Hren et al. 2013), the turnover of terrestrial mammal faunas in Europe known as the Grande Coupure (Stehlin 1909; Schmidt-Kittler and Vianey-Liaud 1975; Brunet 1979) is now considered to be more or less simultaneous with this rapid cooling. A turnover of terrestrial mammal faunas during the Eocene-Oligocene transition is observed in Asia as well (Kraatz and Geisler 2010; Sun et al. 2014). It is now generally accepted that there is a causal relation between the climatic changes and the turnover of terrestrial mammal faunas. The probably late Eocene-early Oligocene south-eastern Serbian faunas show a similar turnover between the faunas of Buštranje-Zvonce and those of the Strelac-Raljin-Valniš (Tables 1 and 2).

The south-eastern Serbian faunas differ from those of supposedly similar age in Central and Western Europe in (1) the presence of a diverse Eocene community of Muridae, (2) the sequence in which the various Muridae subfamilies appear and (3) the consistent presence of a diatomyid and the dipodid *Heosminthus borrae*. Baciu and Hartenberger (2001) were the first to report the presence of cricetids (Muridae) in the late Eocene of Romania.

Heissig (1979), after reviewing the poor Balkan Paleogene, large mammal record suggested that taxa arriving in Central and Western Europe with the Grande Coupure were present well before that time in the Balkan and speculated that immigrants into Europe could have entered through a Balkan migration route.

Later, Nikolov and Heissig (1985) described some large mammal remains from seven upper Eocene and (lower?) Oligocene localities in Bulgaria. Eight species representing seven families were identified. All except one are Asiatic or have strong Asiatic affinities, and only one or two species are possibly ancestors of immigrants into western Europe. This suggests the presence of a barrier for faunal exchange between the Balkan and Central Europe. The Asiatic affinities of the Paleogene large mammals as well as the barrier fit the composition of our rodent faunas from south-eastern Serbia.

Palaeogeographic reconstructions and maps of Europe and adjacent areas have been made by Rőgl (1998), Meulenkamp and Sissingh (2003) and Popov et al. (2004). These reconstructions differ in details, but all show an increase of interconnecting land areas in the region of the Balkan, Asia Minor and Afghanistan during the Oligocene (Figs. 9 and 10).

Comparison of our tentative inventory of the Serbian Paleogene rodent associations, the evidence from the late Eocene beds from Süngülü in the Lesser Caucasus of Anatolia (location shown in Fig. 9; de Bruijn et al. 2003) and the European record suggests that the composition of the rodent assemblages observed cannot be explained by a single migration route without introducing hypothetical ecological corridors and/or filters. In this context, the late Oligocene arrival of *Eucricetodon* and Eomyidae and the presence of Diatomyidae in the Balkan area cannot yet be explained.

## Conclusions

The faunas from south-east Serbia are Eocene and early Oligocene in age. These faunas are located in between Europe and Asia on a potential migration pathway. The Eocene Buštranje fauna contains a diverse Muridae fauna with Pseudocricetdontinae, Paracricetodontinae, Pappacricetodontinae and Melissiodontinae. However, these subfamilies reached Western and Central Europe only during the course of the Oligocene, after the early Oligocene faunal turnover (*Grande Coupure*) of that area.

The association of rodents in the Eocene Buštranje site is unique in containing Pappocricetodontinae, Melissiodontinae, Pseudocricetodontinae and Paracricetodontinae. Species of these subfamilies are clearly more primitive than the Oligocene species of Western and Central Europe. The diversity of the Eocene Paracricetodontinae and the presumably insectivorous Melissiodontinae suggests that these subfamilies originated on the Serbo-Macedonian land area. The presence of diatomyids in the Serbian faunas is the first record of this rodent family outside of Asia.
